# Thulium fiber laser vs Ho:YAG in RIRS: a prospective randomized clinical trial assessing the efficacy of lasers and different fiber diameters (150 µm and 200 µm)

**DOI:** 10.1007/s00345-023-04651-1

**Published:** 2023-10-19

**Authors:** Mark Taratkin, Camilla Azilgareeva, Vladislav Petov, Andrey Morozov, Stanislav Ali, Diana Babaevskaya, Vincent De Coninck, Dmitry Korolev, Gagik Akopyan, Cesare Marco Scoffone, Denis Chinenov, Alexander Androsov, Harun Fajkovic, David Lifshitz, Olivier Traxer, Dmitry Enikeev

**Affiliations:** 1 Institute for Urology and Reproductive Health, Sechenov University, Moscow, Russia; 2 Department of Urology, AZ Klina, Brasschaat, Belgium; 3 Department of Urology, Cottolengo Hospital, Turin, Italy; 4 Sechenov University, Moscow, Russia; 5 Department of Urology, Medical University of Vienna, Währinger Gürtel 18–20, 1090 Vienna, Austria; 6 Karl Landsteiner Institute of Urology and Andrology, Vienna, Austria; 7 Division of Urology, Rabin Medical Center, Petach Tikva, Israel; 8 Sackler School of Medicine, Tel-Aviv University, Tel Aviv, Israel; 9 GRC #20 Lithiase Urinaire, Sorbonne University, Hôpital Tenon, Paris, France

**Keywords:** RIRS, Thulium fiber laser, Ho:YAG

## Abstract

**Introduction:**

The aims of the study: (1) to compare the Super Pulse Thulium Fiber Laser (SP TFL) and the holmium: yttrium–aluminium-garnet (Ho:YAG) lasers in retrograde intrarenal surgery (RIRS); (2) to compare the efficacy of SP TFL laser fibers of different diameters (150 μm and 200 μm).

**Methods:**

A prospective randomized single-blinded trial was conducted. Patients with stones from 10 to 20 mm were randomly assigned RIRS in three groups: (1) SP TFL (NTO IRE-Polus, Russia) with fiber diameter of 150 μm; (2) SP TFL with 200-μm fiber; and (3) Ho:YAG (Lumenis, USA) with 200-μm fiber.

**Results:**

Ninety-six patients with kidney stones were randomized to undergo RIRS with SP TFL using a 150-μm fiber (34 patients) and a 200-μm fiber (32 patients) and RIRS with Ho:YAG (30 patients). The median laser on time (LOT) in the 200-μm SP TFL group was 9.2 (6.2–14.6) min, in 150-μm SP TFL—11.4 (7.7–14.9) min (*p* = 0.390), in Ho:YAG—14.1 (10.8–18.1) min (*p* = 0.021). The total energy consumed in 200-μm SP TFL was 8.4 (5.8–15.2) kJ; 150-μm SP TFL − 10.8 (7.3–13.5) kJ (*p* = 0.626) and in Ho:YAG—15.2 (11.1–25.3) kJ (*p* = 0.005).

**Conclusions:**

Irrespective of the density, RIRS with SP TFL laser has proven to be both a safe and effective procedure. Whilst the introduction of smaller fibers may have the potential to reduce the duration of surgery, SP TFL results in a reduction in the LOT and total energy for stone ablation in RIRS compared with Ho:YAG.

## Introduction

The holmium:yttrium–aluminium-garnet (Ho:YAG) laser used to be the state-of-the-art in lithotripsy. Continuous improvements to this device with pulse modulation and new power possibilities mean that its efficacy has continued to be enhanced year-after-year. Subsequent versions of the laser have incorporated the latest technologies (e.g., pulse modulation). Previous trials were unable to demonstrate any clear advantage over the conventional Ho:YAG [1, 2] laser. They make mention of the fact that we still need more high-quality studies to accurately assess its benefits [3].

The recently introduced SuperPulsed Thulium-fiber laser (SP TFL) has not only already proved to have 2–4 times higher *in-vitro* efficacy in comparison with Ho:YAG and an adequate safety profile [6], but has also been able to demonstrate promising clinical efficacy of lithotripsy in retrograde intrarenal surgery (RIRS) [7]. What is more, the preclinical data show that SP TFL might be used with smaller fibers (fiber diameter of 150 μm) [8] which should not result in lower efficiency but might theoretically increase the irrigation rate thus improving visibility [9, 10]. Recent trials confirm the advantages of TFL over the conventional Ho:YAG [11, 12].

However, there are still no head-to-head comparison data on Ho:YAG with SP TFL lithotripsy in a clinical trial or different laser fiber diameters for SP TFL. Thus, the aims of the study were: 1) to compare SP TFL and Ho:YAG lasers for RIRS and 2) to compare the efficacy of SP TFL using laser fibers (LF) of different diameters (150 μm and 200 μm) for RIRS.

## Materials and methods

Once institutional review board (IRB) approval had been gained, a prospective randomized single-blinded clinical study was conducted (Sechenov University, Russia) according to the Consolidated Standards of Reporting Trials (CONSORT) guidelines (ClinicalTrials.gov database registration ID – NCT04346485).

### Design

Once informed consent was obtained, patients with kidney stones from 10 to 20 mm were randomly assigned to undergo RIRS using a SP TFL (NTO IRE-Polus, Russia) laser with a reusable LF diameter of 150 μm or 200 μm or a Ho:YAG laser (Lumenis, USA) with a 200-μm reusable LF at a 1:1:1 ratio. Exclusion criteria were multiple (more than three) kidney stones over 5 mm in diameter; ureteral stones; anticoagulant or antiplatelet therapy, the need for secondary simultaneous surgical intervention (for benign prostatic hyperplasia, upper tract carcinoma, urethral and ureteral stricture), and history of previous stone surgery or SWL.

Based on a non-inferiority sample size calculation, in total 87 patients (29 per group) should have been included in the study for an 80% statistical power with the upper limit of a one-sided 95% confidence interval exceeding a > 5% difference in favor of the standard treatment group. Bearing in mind a drop-off rate of 20%, 105 participants were included (35 patients in each group). For the randomization of the patients, a computer‐generated allocation sequence was used. Patients were assigned treatment on a masked basis. An online system for randomization was used (https://www.studyrandomizer.com/). As we were trying to exclude all the confounding factors, we stratified patient randomization based on the surgeon and stone location to balance treatment arms.

The primary objective of the study was to evaluate the efficiency of lithotripsy using fibers with different diameters, measured through the following parameters: laser on time (LOT), ablation speed (mm^3^/sec), ablation efficacy (J/mm^3^), and energy consumption (J/sec). Secondary outcomes were radiation exposure time, operation time, complication rates, blood loss, duration of catheterization and stone-free rate (SFR).

### Assessments

Prior to inclusion in the study, all the patients underwent contrast-enhanced computed tomography (CT) to assess stone size and medium density (HU) [13]. The approximate stone volume was calculated using the ellipsoid volume formula (4/3 × π × radius length × radius width × radius depth).

All the data were recorded by a single researcher. Stone size, stone volume, density, laser-on time (LOT, total time of laser ablation, automatically measured and recorded by the laser machine), total energy for stone ablation (automatically measured and recorded by the laser machine), operation time (from instrument insertion until removal), fluoroscopy time, radiation exposure, catheterization time, and hemoglobin level prior to and after surgery were measured. Where the patient had several stones (2 or 3), the total volume was calculated using the sum of the volumes of each stone. The following parameters were also calculated: stone ablation speed (stone volume/LOT, mm^3^/sec), lithotripsy efficacy (total energy for stone ablation/stone volume, J/mm^3^) and energy consumption efficacy (total energy for stone ablation/LOT, J/sec). Visibility was assessed using the surgeon reported visibility score (0—sufficient visibility, 1—minor decrease, 2—poor visibility necessitating regimen change).

The postoperative stone-free rate as well as the subsequent complication rates (e.g., hydronephrosis) were assessed using low-dose CT three months after surgery. Patients without residual fragments, or with fragments smaller than 2 mm on CT at 3 months, were considered to be stone-free. Post-operative complications were scored according to the Clavien–Dindo classification [14].

### Surgery

The procedures were performed by 5 surgeons highly experienced in laser lithotripsy (who had performed at least 50 procedures prior to the start of this study). According to local clinical practice, all patients were “pre-stented” 4–7 days before the procedure, prior to the subsequent use of a ureteral access sheath (12–14 Fr, Cook, USA, and 11–13 Fr, Navigator HD, Boston Scientific, Marlborough, USA). RIRS was performed under general anesthesia in the lithotomy position. The lithotripsy settings were based on previous research completed on the topic [15]. During the procedure, the LithoVue™ (Boston Scientific, Marlborough, USA) flexible ureteroscope was used. In two patients, the FLEX-X2 (Karl Storz GmbH, Germany) ureteroscope was used. Intraoperative irrigation was performed using Traxer flow [16] in all patients.

According to our previous research, the most efficient regimens for stone ablation were suggested (fragmentation (1.5 J × 20 Hz), dusting (0.5 J × 30 Hz) or popcorning (0.15 J × 100 Hz)). However, surgeons could adapt the regimen to their own needs if necessary [15]. Similar regimens were used in all devices. At the end of the procedure, a ureteral JJ stent was placed for up to 14 days postoperatively, according to the accepted practice in our institution. The bladder was drained using a Foley catheter for the duration of at least one day.

### Statistical analysis

We used SPSS Statistics 23.0 (IBM, USA) for statistical analysis. Mean and standard deviation (SD) values, median and quartile distributions were calculated for variables. The Shapiro–Wilks test was used for distribution assessment, Student’s *t*-test or Kolmogorov–Smirnov, one-way ANOVA and Mann–Whitney *U* tests were used where appropriate. A *p*-value of 0.05 was chosen as the threshold of statistical significance.

## Results

**Fig. 1:**
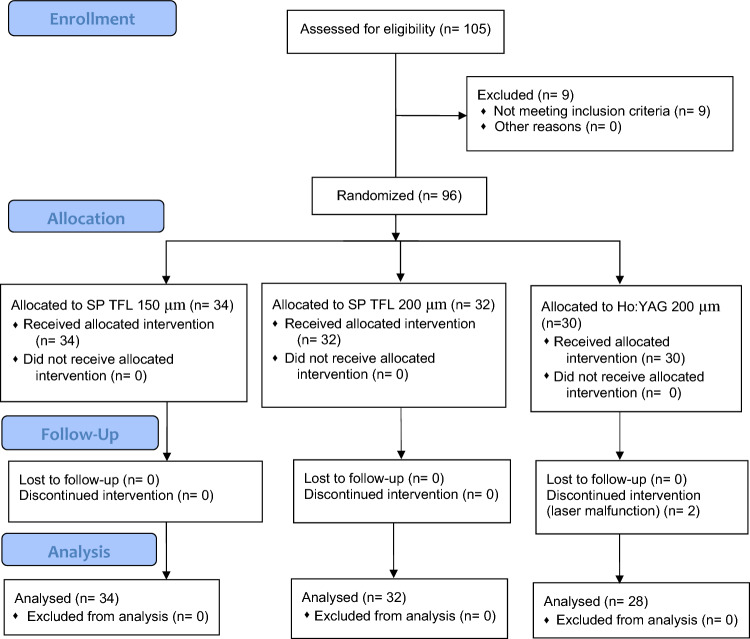
CONSORT flowchart

Initially 116 patients were identified to be enrolled in the study. Eleven of them rejected participation. Thereafter 9 of 105 enrolled patients were excluded as they were unable to participate. Between January 2020 and October 2021, the remaining 96 patients were randomized into three groups: RIRS with SP TFL using a 150-μm LF (34 patients) and a 200-μm LF (32 patients), and RIRS with Ho:YAG (30 patients). Exact data on enrollment, exclusion and allocation are present in the CONSORT flowchart (Fig. 1). The groups were similar in terms of mean stone density (1029 ± 340 HU in the 150-μm LF group, 1155 ± 364 HU in the 200-μm LF group and 1094 ± 350 HU in the Ho:YAG group, *p* = 0.921) and median stone volume (361.3 mm^3^ (200.3–628.3) in the 150-μm LF group, 329.9 mm^3^ (293.2–628.3) in the 200-μm LF group and 508.9 mm^3^ (293.2–565.5) in the Ho:YAG group, *p* = 0.685). Locations of the stones were similar between the groups: 150-μm SP TFL (14—lower calyx, 13—renal pelvis, 7—upper calyx); 200-μm SP TFL (13—lower calyx, 11—renal pelvis, 8—upper calyx); Ho:YAG (10—lower calyx, 12—renal pelvis, 8—upper calyx). It should be noted that in two cases (one in the 150-μm TFL group and one in the Ho:YAG group) we used a FLEX-X2 ureteroscope (Karl Storz GmbH, Germany).

The median LOT was minimal in the 200-μm SP TFL group [9.2 (6.2–14.6) min]. Similar results were obtained in the 150-μm SP TFL group [11.4 (7.7–14.9) min, *p* = 0.390]. Greater LOT was found in Ho:YAG [14.1 (10.8–18.1) min, *p* = 0.021]. Similar findings were observed in terms of median total energy: 200-μm SP TFL (8.4 (5.8–15.2) kJ; 150-μm SP TFL [10.8 (7.3–13.5) kJ, *p* = 0.626] and Ho:YAG [15.2 (11.1–25.3) kJ, *p* = 0.005] (Table 1, 2).

**Table 1:** Patient characteristics, intraoperative and postoperative data for RIRS with SP TFL using 150- and 200-μm fibers

	SP TFL 150 μm *N* = 34	SP TFL 200 μm *N* = 32	*p*-value
Mean age, years (range)	54.5 ± 12.2 (33–78)	51.2 ± 14.1 (28–83)	0.435
Mean stone size, mm ± SD (range)	11.3 ± 3.2 (6.0–20.0)	11.4 ± 3.1 (5.0–20.0)	0.895
Median stone volume, mm^3^ (IQR)	361.3 (200.3–628.3)	329.9 (293.2–628.3)	0.493
Mean stone density, HU ± SD (range)	1029 ± 340 (320–1500)	1154 ± 364 (360–1940)	0.808
Median laser on time, min (IQR)	11.4 (7.7–14.9)	9.2 (6.2–14.6)	0.390
Median total energy for stone ablation, kJ (IQR)	10.8 (7.3–13.5)	8.4 (5.8–15.2)	0.626
Median ablation speed, mm3/sec (IQR)	0.7 (0.5–1.0)	0.8 (0.5–1.0)	0.447
Median ablation efficacy, J/mm3 (IQR)	27.4 (17.3–32.8)	24.5 (14.5–32.3)	0.713
Median energy consumption, J/sec (IQR)	15.2 (15.0–18.6)	17.1 (15.0–24.2)	0.111
Mean operation time, min ± SD (range)	60.2 ± 20.1 (20–105)	74.4 ± 26.3 (35–120)	0.044*
Median fluoroscopy time, sec (IQR)	51.0 (32.0–78.0)	56.0 (40.0–119.0)	0.355
Median radiation exposure, mGy/cm^2^ (IQR)	350.5 (222.0–526.9)	328.5 (121.0–898.5)	0.918
UAS use ratio: 11–13 Fr, N	19 / 34	19 / 32	0.485
12–14 Fr, N	15 / 34	13/ 32	
Mean catheterization time, days ± SD	1.5 ± 1.4	1.7 ± 1.5	0.579
Mean preoperative Hb, mmol/l ± SD	143.0 ± 16.3	143.4 ± 15.3	0.782
Mean postoperative Hb, mmol/l ± SD	139.0 ± 16.4	137.3 ± 18.9	0.436

Data presented as mean ± SD (range) or median (IQR) where appropriate

^*^Statistically significant difference with *p* < 0.05

**Table 2:** Patient characteristics, intraoperative and postoperative data for RIRS using SP TFL and Ho:YAG with 200-μm fibers

	SP TFL 200 μm (*n* = 32)	Ho:YAG 200 μm (*n* = 28)	*p*-value
Mean age, years (range)	51.2 ± 14.1 (28–83)	53.4 ± 15.1 (30–81)	0.551
Mean stone size, mm ± SD (range)	11.4 ± 3.1 (5.0–20.0)	12.3 ± 3.9 (7.0–20.0)	0.477
Median stone volume, mm^3^ (IQR)	329.9 (293.2–628.3)	508.9 (293.2–565.5)	0.236
Mean stone density, HU ± SD (range)	1155 ± 364 (360–1940)	1094 ± 350 (400–1500)	0.806
Median laser on time, min (IQR)	9.2 (6.2–14.6)	14.1 (10.8–18.1)	0.021*
Median total energy for stone ablation, kJ (IQR)	8.4 (5.8–15.2)	15.2 (11.1–25.3)	0.005*
Median ablation speed, mm3/sec (IQR)	0.8 (0.5–1.0)	0.6 (0.4–0.9)	0.187
Median ablation efficacy, J/mm3 (IQR)	24.5 (14.5–32.3)	31.8 (22.5–42.5)	0.046*
Median energy consumption, J/sec (IQR)	17.1 (15.0–24.2)	20.0 (15.0–25.8)	0.699
Mean operation time, min ± SD (range)	74.4 ± 26.3 (35–120)	90.0 ± 31.8 (35–165)	0.054
Median fluoroscopy time, sec (IQR)	56.0 (40.0–119.0)	65.0 (58.0–107.0)	0.241
Median radiation exposure, mGy/cm^2^ (IQR)	328.5 (121.0–898.5)	377.0 (226.8–885.3)	0.366
UAS use ratio: 11–13 Fr, N	19 / 32	18 / 10	0.451
12–14 Fr, N	13 / 32	18 / 10	
Mean catheterization time, days ± SD	1.7 ± 1.5	1.1 ± 0.3	0.075
Mean preoperative Hb, mmol/l ± SD	143.4 ± 15.3	138.1 ± 18.7	0.238
Mean postoperative Hb, mmol/l ± SD	137.3 ± 18.9	132.8 ± 18.2	0.998

Data presented as mean ± SD (range) or median (IQR) where appropriate

^*^Statistically significant difference with *p* < 0.05

The median ablation speed was 0.7 (0.5–1.0) mm^3^/sec for 150-μm SP TFL, 0.8 (0.5–1.0) mm^3^/sec for 200-μm SP TFL and 0.6 (0.4–0.9) for Ho:YAG (*p* = 0.472). The median ablation efficacy for 200-μm SP TFL (24.5 (14.5–32.3) J/ mm^3^) was similar to 150-μm SP TFL (27.4 (17.3–32.8) J/ mm^3^, p = 0.713), but lower in comparison to Ho:YAG (31.8 (22.5–42.5) J/mm^3^, *p* = 0.046**)**. The median energy consumption was similar in all groups: 15.2 (15.0–18.6) J/sec for 150-μm LF, 17.1 (15.0–24.2) J/sec for 200-μm LF and 20.0 (15.0–25.8) J/sec for Ho:YAG (*p* = 0.140).

The duration of the surgery was shortest in the 150-μm SP TFL group (60.2 ± 20.1 min), and this was shorter than in the 200-μm SP TFL group (74.4 ± 26.3 min, *p* = 0.044). Both durations were shorter than that in the Ho:YAG group (90.0 ± 31.8 min, *p* = 0.006). Impaired visibility was found in 2 patients in the 150-μm SP TFL group, 5 patients in the200-μm SP TFL group (*p* = 0.18) and 7 patients in the Ho:YAG group (*p* = 0.049).

The mean catheterization time was comparable among groups (1.5 ± 1.4 days for 150-μm SP TFL, 1.7 ± 1.5 days for 200-μm SP TFL and 1.1 ± 0.3 days for Ho:YAG (*p* = 0.212).

The median fluoroscopy time did not differ between groups [51.0 (32.0–78.0) sec for 150-μm SP TFL, 56.0 (40.0–119.0) sec for 200-μm SP TFL and 65.0 (58.0–107.0) sec for Ho:YAG, *p* = 0.852]. The median radiation exposure was also similar between groups [350.5 (222.0–526.9) mGy/cm^2^ for 150-μm SP TFL, 328.5 (121.0–898.5) mGy/cm^2^ for 200-μm SP TFL and 377.0 (226.8–885.3) mGy/cm^2^ for Ho:YAG, *p* = 0.959] (Table 1, 2).

At the 3-month follow-up, no large stone fragments (> 2 mm) were observed in any of the patients. The postoperative complication rate was low, with grade I–II Clavien–Dindo complications present in 2 (2.1%) patients: 1 patient from the 150-μm SP TFL group had a fever (Grade I) and 1 patient from the 200-μm SP TFL group had UTI (Grade II). No complications specific to the use of SP TFL were identified.

## Discussion

The current trial is the first RCT that compares RIRS with Ho:YAG and SP TFL. Whilst it was shown that both lasers are equally safe and effective, the SP TFL (with a smaller fiber, or without) resulted in a shorter LOT and used nearly half the amount of total energy for stone ablation as Ho:YAG. The mean operation time tended to be lower in the SP TFL group compared to the Ho:YAG. However, the difference was not statistically significant.

In the recently published trial by Ulvik et al. SP TFL produced better SFR than Ho:YAG (92% vs 67%) [17]. Our study results were different and this might be due to the different approach that we adopted (pre-stenting, standard hospital stay instead of a single-day hospital stay). However, we found similar results in terms of operation time with SP TFL performing better as well as similar rate of impaired visibility in the smaller fiber group (5.8% vs 23%, *p* = 0.049).

The previous preclinical studies assumed that lithotripsy efficiency of TFL was superior to that of Ho:YAG. In an *in-vitro* study on the effects of pulse shape by Ventimiglia et al., SP TFL showed 1.8–2.2 times higher ablation efficiency measured by stone ablation volume than Ho:YAG [18]. Moreover, ablation efficacy—the parameter indicating the energy required to ablate a stone—was lower in the SP TFL group than in Ho:YAG group, meaning that SP TFL requires less energy per single ablation.

Unfortunately, our trial was conducted comparing SP TFL with a conventional Ho:YAG without pulse modulation. Majdalany et al. in their clinical study of pulse-modulated Ho:YAG in RIRS concluded that fragmentation speed (or ablation speed) was the most suitable parameter for measuring laser efficacy. In their study, pulse-modulated Ho:YAG had shown a mean fragmentation speed of 0.9 mm^3^/s [1]. In the current study, we found that median ablation speed was 0.8 (0.5–1.0) mm^3^/sec for 200-μm SP TFL, 0.7 (0.5–1.0) mm^3^/sec for 150-μm SP TFL and 0.6 (0.4–0.9) for Ho:YAG (*p* = 0.187).

As for the smaller fiber, the results of the trial are quite interesting. Whilst the use of a smaller fiber may result in additional efficacy due to its ability to create a smaller stone fragment (as suggested by Panthier et al. [19]), small core fibers are also able to increase the flexibility of ureteroscope tip deflection and improve irrigation. All this allows for better visualization of the stone.

Our study was also the first clinical comparison between 150-μm and 200-μm SP TFL fibers. We were able to show that there are no differences in performance parameters, except for the shorter operation time for smaller LF (60.2 ± 20.1 min and 74.4 ± 26.3 min, respectively, *p* = 0.044). One important finding is that TFL operated with a similar efficiency to Ho:YAG until the fiber diameters were switched. There are a few possible explanations for this. Firstly, a smaller fiber would certainly lead to better water flow and thus better visibility, shortening surgery. Secondly, it is known that SP TFL is characterized by a gaussian shaped (U-shaped) spatial beam profile with a small diameter of 50–70 μm (200 μm for Ho:YAG). Previously, it was suggested that this may lead to several advantages: smaller dust formation and better ablation efficacy [10, 19]. Using a smaller core diameter fiber in the SP TFL increases the beam density fourfold by reducing the beam focal spot and using less fiber diameter. This results in smaller bubble formation leading to effectivelithotripsy with reduced retropulsion, potentially better irrigation rates and sufficient visibility. Thirdly, the current study was designed to detect differences in ablation speed between the lasers. Further assessment is necessary to confirm the superiority of smaller fibers with respect to operative time. A previous *in-vitro* study comparing SP TFL using 150-μm LF with a 200-μm LF performed by Taratkin et al. suggested no inferior efficacy of smaller LF. This was subsequently proved in the clinical setting [8].

A 3 months, low-dose computed tomography showed that 100% of patients were stone free, with no patients showing a residual stone fragment of ≥ 3 mm which is comparable to the SFR reported in the literature [20–25]. Also, no cases of severe complications were recorded. Only two patients showed grade I or II Clavien–Dindo complications, and no laser-specific complications were identified.

Our study is not without its limitations. The composition of stones was not presented in our trial, but the stone density assessed in all groups meant that we could evaluate laser efficacy in various types of stones. Moreover, it was shown that TFL is effective in producing stone dust from all stone types [26, 27]. In the current study, we did not assess retropulsion caused by lasers. However, we believe that previous *in-vitro* and clinical data have clearly shown comparable retropulsion rates between TFL and Ho:YAG and between different TFL fiber diameters [7, 28]. The procedures were performed both with reusable and single-use scopes. However, we believe this did not influence the outcomes as previous clinical trials suggest [29]. Pre-stenting which is not necessarily recommended in the guidelines, is a usual practice in our institute and was carried out on all the patients in our trial. Also, a meta-analysis performed by Law et al. concluded that pre-stenting may improve the overall SFR and decrease intraoperative ureteric injury [30]. Our study did not aim to assess the cost-effectiveness of reusable fibers. Given that a previous *in-vitro* experiment had demonstrated that the fiber burnback was equivalent for both holmium and thulium fiber lasers, we found no need to re-evaluate this point. Despite the absence of any statistically significant difference (*p* = 0.236), the holmium laser stone group volume was almost greater than that of thulium groups which also can be a potential source of bias.

Also, the Ho:YAG device used in the trial was a previous-generation machine without pulse modulation. We do not see this issue as a major limitation to pulse modulation for it has not proven to have superior efficacy in clinical trials. We also would like to note that the described settings (disposable instruments and LF, duration of post operation double J-stent placement, etc.) cannot be generalized to each clinical center.

## Conclusion

RIRS with SP TFL laser is a safe and effective procedure, irrespective of the stone density. Whilst the introduction of smaller fibers could result in a shorter surgery duration, SP TFL can decrease the LOT and total energy for stone ablation in RIRS compared to Ho:YAG. The stone-free rate and complication rates are comparable for both lasers.

## Data Availability

On demand.

## Abbreviations

Ho:YAG: Holmium:YAG
SP TFL: SuperPulsed thulium fiber laser
RIRS: Retrograde intrarenal surgery
LOT: Laser on time
CT: Computed tomography
LF: Laser fiber
EEP: Endoscopic enucleation of the prostate
